# PI3K/AKT/mTOR pathway and its related molecules participate in PROK1 silence-induced anti-tumor effects on pancreatic cancer

**DOI:** 10.1515/biol-2022-0538

**Published:** 2023-04-10

**Authors:** Feng Wang, Xiaogang Yan, Yongqiang Hua, Jianjun Song, Di Liu, Chun Yang, Fei Peng, Fuping Kang, Yongfeng Hui

**Affiliations:** Department of Hepatobiliary Surgery, General Hospital of Ningxia Medical University, No. 804 South Shengli Street, Xingqing District, Yinchuan 750001, Ningxia, China; Ningxia Clinical Medical Research Center of Hepatobiliary and Pancreatic Surgical Diseases, Yinchuan 750001, China; Department of Surgical Oncology, The First People’s Hospital of Yinchuan, Yinchuan 750001, China; Minimally Invasive Treatment Center, Fudan University Shanghai Cancer Center, Shanghai 200032, China; Department of Oncology, Shanghai Medical College, Fudan University, Shanghai 200032, China; Department of Colorectal Surgery, General Hospital of Ningxia Medical University, Yinchuan 750001, China; Department of Hepatobiliary Pancreatic Surgery, Edong Healthcare Huangshi Central Hospital, Huangshi 435002, Hubei, China

**Keywords:** pancreatic cancer, PROK1, apoptosis, proliferation, PI3K/AKT/mTOR

## Abstract

The PI3K/AKT/mTOR (phosphatidylinositol 3-kinase/protein kinase B/mammalian target of rapamycin) pathway can be initiated by PROK1 (prokineticin 1), but its effect and mechanism of action in pancreatic carcinoma (PC) are not fully understood. In this study, we elucidated the roles of PROK1 and its related molecules in PC *in vivo*. PANC-1 cells with PROK1 knockdown were injected into BALB/c nude mice. The growth and weight of the tumor were monitored and measured, which was followed by TUNEL (terminal deoxynucleotidyl transferase biotin-dUTP nick end labeling), immunohistochemical staining, and hematoxylin and eosin staining. The key proteins related to proliferation, apoptosis, and the PI3K/AKT/mTOR pathway were determined by Western blotting. We also used public databases to identify the molecules related to PROK1. The reduction of PROK1 inhibited angiopoiesis and promoted apoptosis *in vivo*. PCNA-1, cyclin D1, and Bcl-2 decreased considerably, while Bax and cleaved caspase-3 increased significantly after PROK1 inhibition. The PI3K/AKT/mTOR signal inhibition was also closely associated with PROK1 knockdown. The possible related molecules of PROK1, such as von Willebrand factor, were screened and considered to be involved in the aberrant activation of PI3K/AKT. In conclusion, PROK1 knockdown significantly prevented tumor growth and promoted apoptosis of human PC cells *in vivo*, where the PI3K/AKT/mTOR pathway was probably inhibited. Therefore, PROK1, along with its related molecules, might be important targets for PC therapy.

## 1 Introduction

Pancreatic carcinoma (PC) occurs in the digestive system. It has a high degree of malignancy and leads to more than 460,000 deaths worldwide in 2020 [1]. PC ranks fourth in the United States and other developed countries for cancer-related mortality. The PC mortality rate in China is 2.62/100,000 and ranks tenth for malignant tumor-related death [2]. The incidence of PC is increasing every year worldwide. Although surgery is commonly performed for treating PC, it is not specific to the early stage of the disease. The cancer cells can infiltrate and metastasize at an early stage of cancer; less than 20% of the patients with PC can be treated by surgical resection [3]. PC has an extremely poor prognosis, even in those who undergo surgery, and the incidence of complications even after an operation and local or regional recurrence remains high. The average survival time of PC patients is less than 6 months, the 5-year survival rate is < 5%, and 90% of the patients who do not undergo surgery die within 1 year [4].

Angiogenesis induced by cancer, including PC, promotes tumor growth, invasion, and metastasis. Proangiogenic factors stimulate vascular endothelial cell proliferation and angiogenesis [5]. PROK1 (prokineticin 1), also known as EG-VEGF, is the first tissue-specific proangiogenic factor that was found to contract gastrointestinal smooth muscles and promote intestinal motility. High levels of PROK1 are found in steroid hormone-producing organs, such as the ovary, testes, adrenals, and placenta, and it participates in the development of tumors in various specific and related tissues [6]. PROK1 is diffusely distributed in PC tissues, with a positive expression rate of up to 95%, indicating that PROK1 and the occurrence and development of PC might be correlated [7,8].

The PI3K/AKT/mTOR (phosphatidylinositol 3-kinase/protein kinase B/mammalian target of rapamycin) pathway is an important signaling pathway in cells and can be activated by PROK1. The pathway participates in cell growth, proliferation, apoptosis, and also various pathological processes of human cancer [9]. PI3K is an upstream signaling molecule of the PI3K/AKT/mTOR pathway and is a key member of the phospholipase kinase family with lipid and protein kinase activity [10]. PI3Ks are mainly of three types, but only PI3K1 is closely related to tumorigenesis [11]. AKT is an important downstream kinase in the PI3K/AKT/mTOR signaling pathway, and it can be converted to phosphorylated AKT by the PI3K-mediated activation of the growth factor receptor. The phosphorylated AKT (p-AKT) can activate multiple downstream signaling molecules [12], inhibit tumor cell autophagy through mTOR, phosphorylate MADD, caspase-3, caspase-9, phosphorylate FOXO-1 to control the cell cycle, and promote tumor cell growth and/or metastasis [13,14]. mTOR is one of the main downstream signaling molecules and regulates cyclin D1, Bad, and Bcl-2. To summarize, the PI3K/AKT/mTOR pathway promotes tumor cell proliferation, migration, and angiogenesis [15]. The PI3K/AKT/mTOR pathway is continuously activated in most PC cell lines [16,17].

In this study, we transfected a specific lentivirus-mediated short hairpin RNA (shRNA) into pancreatic cells and studied the effect of PROK1 silencing on PC *in vivo* by monitoring the growth of the tumor, determining cell apoptosis, detecting the protein level of proliferation markers and anti-apoptotic markers, and measuring the activation level of the key molecules of the PI3K/AKT/mTOR signal.

## 2 Methods

### 2.1 Animals

Male BALB/c nude mice (Inbred strain of mouse, 6 weeks old) were obtained from Shanghai SLAC Laboratory Animal Co. Ltd. (China). The mice were kept under specific-pathogen-free conditions and provided with enough food, water, and space.


**Ethical approval:** The research related to animal use has been complied with all the relevant national regulations and institutional policies for the care and use of animals, and was approved by the Ethics Committee of the General Hospital of Ningxia Medical University (approval number: KYLL-2022-0628).

### 2.2 shRNA-mediated knockdown of PROK1

The steps for the shRNA-mediated knockdown of PROK1 were the same as those followed in our previous study [8]. ShPROK1 was used to transfect PANC-1 cells to knock down PROK1 expression. The shRNA sequences for shPROK1 and the negative control (shCon) were designed and synthesized for the vector pYr-1.1-hU6-EGFP-Neo (Catalog No. #VRY0359, YRbio; Changsha, China). The shPROK1 sequence is as follows: 5′-CACCCTCCATGGACTTGAAGAACATCTCGAGATGTTCTTCAAGTCCATGGAGTTTTTTG-3′ (underlined: sequence target at PROK1). The negative control sequence is as follows: 5′-CACCCCTAAGGTTAAGTCGCCCTCGCTCGAGCGAGGGCGACTTAACCTTAGGTTTTTTG-3′ (underlined: sequence target at no gene). Before lentivirus packaging, the BsaI restriction enzyme (Dalian TAKARA Co. Ltd., China) was used to digest the shuttle plasmid, and then, T4 ligase was used to connect the pYr-1.1 fragments and anneal the double-stranded shRNA. The recombined lentivirus was used to infect the cells when cell confluence reached 80–90%. After 72 h, the supernatant was collected, centrifuged at 4°C, filtered with a filter (0.45 µm), and finally, the product was collected. Then, the cells were transferred to six-well plates and incubated at 37°C with 5% CO_2_ for 24 h. In the presence of polybrene (6 µg/mL), the cells were incubated with shPROK1. A scramble sequence, designated as shCon, was ligated into the vector and served as a negative control. After 24 h, the infected cells were screened out using puromycin, and the cells were harvested to assess the knockdown of PROK1.

### 2.3 Establishing a nude mouse xenograft model bearing human PC cells

Male BALB/c nude mice (6 weeks old; *n* = 10) were randomly divided into two groups. The mice in one group were injected with 2 × 10^6^ control PANC-1 cells (shCon), and those in the other group were injected with PANC-1 cells transfected with sh-PROK1–1. The cell suspension (2 × 10^6^ cells/mL, 200 µL) containing shPROK1 in PBS was injected into the right armpits, and the PC tumors were allowed to grow in the mice for 49 days. The tumor volume was measured using calipers, and the weight of all animals was recorded throughout the experiment. The tumor volume was calculated using the formula: tumor volume = length (cm) × width (cm^2^) × 0.5236. On Day 49, the mice were euthanized. The tumor tissue was macroscopically observed after execution and prepared for histopathology and biochemical analysis.

### 2.4 Immunohistochemical (IHC) staining

Subcutaneous PC tumor masses were used for the IHC analysis to detect the expression of PROK1 in human PC xenograft in BALB/c nude mice. The prepared sections (4 µm) were dry-roasted and dehydrated in an oven at 65°C for 30 min and then were dewaxed in xylene, dehydrated in alcohol, and finally immersed in a solution tank containing double distilled water for 10 min. The sections were immersed in 3% H_2_O_2_ for 10 min, washed in distilled water thrice for 2 min, and immersed in 0.01 M tannic acid repair solution. Finally, they were heated in a microwave oven for 10 min at 92–98°C. The sections were placed in a glass jar containing 3% hydrogen peroxide and incubated at room temperature for 15 min to eliminate endogenous peroxidase activity. Normal sheep serum blocking solution was added dropwise to the sections, which were then incubated at 37°C for 30 min. Then, the excess liquid was removed from the sections. The primary antibody against PROK1 (Santa Cruz Biotechnology; sc-30343; dilution, 1:50) or PCNA (Cell Signaling Technology; #13110; dilution, 1:10,000) was added to the sections, which were then placed in a humidified space at 4°C overnight. The sections were taken out of the refrigerator, rewarmed at 37°C for 30 min, and washed thrice with PBS buffer for 5 min. The biotin-labeled secondary antibody was then added dropwise to the sections and incubated at 37°C for 30 min. The sections were stained using the Histostain-Plus Generation 3 IHC Assay Kit (Invitrogen, USA) following the manufacturer’s protocol. The positively stained cells were counted (for PCNA) or densitometrically analyzed (for PROK1) in five views under a microscope.

### 2.5 TUNEL (terminal deoxynucleotidyl transferase-mediated nick end labeling)

The paraffin sections to be dyed were baked in an oven at 60°C for 3 h, dewaxed with xylene, and rehydrated using gradient alcohol. After adding proteinase K, the sections were incubated at 37°C for 8 min, followed by the addition of 0.3% hydrogen peroxide in methanol and incubation for 10 min at room temperature to block endogenous peroxide enzymes. Then, 0.1% (v/v) Triton–sodium citrate was added to the sections for 10 min for cell penetration and antigen retrieval. The TUNEL reaction mixture solution was added and incubated at 37°C in a dark box for 1.5 h. Next, the transformant-POD was added and incubated in the wet box for 30 min at 37°C. After the DAB substrate solution was added, the samples were incubated at room temperature for 1–2 min. The sections were counterstained with hematoxylin for 30 s and differentiated with 1% hydrochloric acid alcohol for 5 s. Then, the sections were dehydrated with alcohol, treated with xylene, covered with a coverslip, and sealed with neutral gum. Finally, the sections were analyzed under a confocal laser scanning microscope.

### 2.6 Western blot analysis

The tumor tissue collected from the nude mice was ground to a granular shape with liquid nitrogen in a mortar. The tissue was placed in a 1.5 mL centrifuge tube. To 100–200 µL of the cell lysate, a pre-warmed 1× SDS gel-loading buffer was added as soon as possible. The sample was placed in a boiling water bath for 5 min. Then, the sample was centrifuged at 10,000 rpm at room temperature. The BCA Protein Assay Reagent (Millipore, Bedford; MA, USA) was used to quantify the extracted protein. The collected proteins (30 mg) were separated by 8% SDS-PAGE and transferred onto a PVDF membrane. After blocking the membrane with non-fat milk (5%) for 2 h, the primary antibodies against the following antigens were incubated with the membrane overnight at 4°C for 24 h: PROK1 (Santa Cruz Biotechnology; sc-30343; dilution, 1:300), PCNA (CST; #13110; dilution, 1:2,000), cyclin D1 (CST; #55506; dilution, 1:2,000), Bax (CST; #2772; dilution, 1:1,000), Bcl-2 (CST; #4223; dilution, 1:1,000), cleaved caspase-3 (CST; #9654; dilution, 1:1,000), PI3K (Abcam; ab154598; dilution, 1:2,000), phospho-PI3K [Tyr458] (Abcam; ab278545; dilution, 1:2,000), AKT (Abcam; ab8805; dilution, 1:500), phospho-AKT [Ser473] (Abcam; ab81283; dilution, 1:5,000), mTOR (Abcam; ab32028; dilution, 1:2,500), and phospho-mTOR [Ser2448] (Abcam; ab109268; dilution, 1:5,000). The samples were incubated with horseradish peroxidase-conjugated secondary antibodies at room temperature for 2 h. The signals were detected using an ECL system (Amersham Biosciences, USA). Densitometric analysis of the blots was performed using the ImageJ software.

### 2.7 Histological analysis

The samples were fixed with neutral formalin solution (10%) for 1 day and rinsed. Increasing concentrations of ethanol were used to dehydrate the samples. The tissues were embedded in paraffin and cut into 4 µm sections using a microtome. A graded descending ethanol series, including 100, 90, and 70% ethanol solutions (5 min each) and dH_2_O (10 min), were used to deparaffinize and rehydrate the sections successively. Finally, the sections were stained with hematoxylin and eosin (H&E) using the conventional method.

### 2.8 Public data mining

We further determined the related molecules that regulate PROK1 or are regulated or affected by PROK1 using GEPIA 2 (http://gepia2.cancer-pku.cn/) in the next-generation sequencing databases TCGA (https://portal.gdc.cancer.gov/) and GTEx (https://www.gtexportal.orghome/). The expression level of PROK1 and its possible related genes (von Willebrand factor [VWF], GNG11, FGF7, TNXB, IL3RA, and LPAR1) in PAAD (pancreatic adenocarcinoma) and the data from paired adjacent tissue or normal pancreas tissue served as the control. The association between the expression level of the genes and the survival of the patients was analyzed. Initially, the possible related genes were screened out (criteria: Pearson’s correlation coefficient >0.3 and top 500 genes) from those that were significantly correlated with PROK1 in the PAAD dataset from the TCGA database using the tools “Similar Genes Detection” on GEPIA 2 and then were assessed by the KEGG enrichment analysis (WebGestalt, http://www.webgestalt.org/). We selected the genes enriched in the PI3K/AKT signaling pathway and overlapped them with all differentially expressed genes (DEGs, cutoff: |Log_2_FC| = 1, *p* < 0.01) in PAAD. The protein levels of VWF, GNG11, FGF7, and IL3RA were obtained from THPA (https://www.proteinatlas.org/), and survival analysis was performed using the Kaplan–Meier plotter (https://kmplot.com/analysis/) or ToPP (http://www.biostatistics.online/topp/index.php) for univariate or multivariate factors.

### 2.9 Statistical analysis

Statistical analyses were performed using the GraphPad Prism software (version 8.0). All data are presented as mean ± SD (standard deviation) from at least three replicates. The differences among groups were determined by performing a one-way analysis of variance (ANOVA) followed by the least significant difference test. All differences among and between groups were considered to be statistically significant at *P* < 0.05.

## 3 Results

### 3.1 PROK1 knockdown decreased the volume and weight of human PC xenografts in BALB/c nude mice

**Figure 1:**
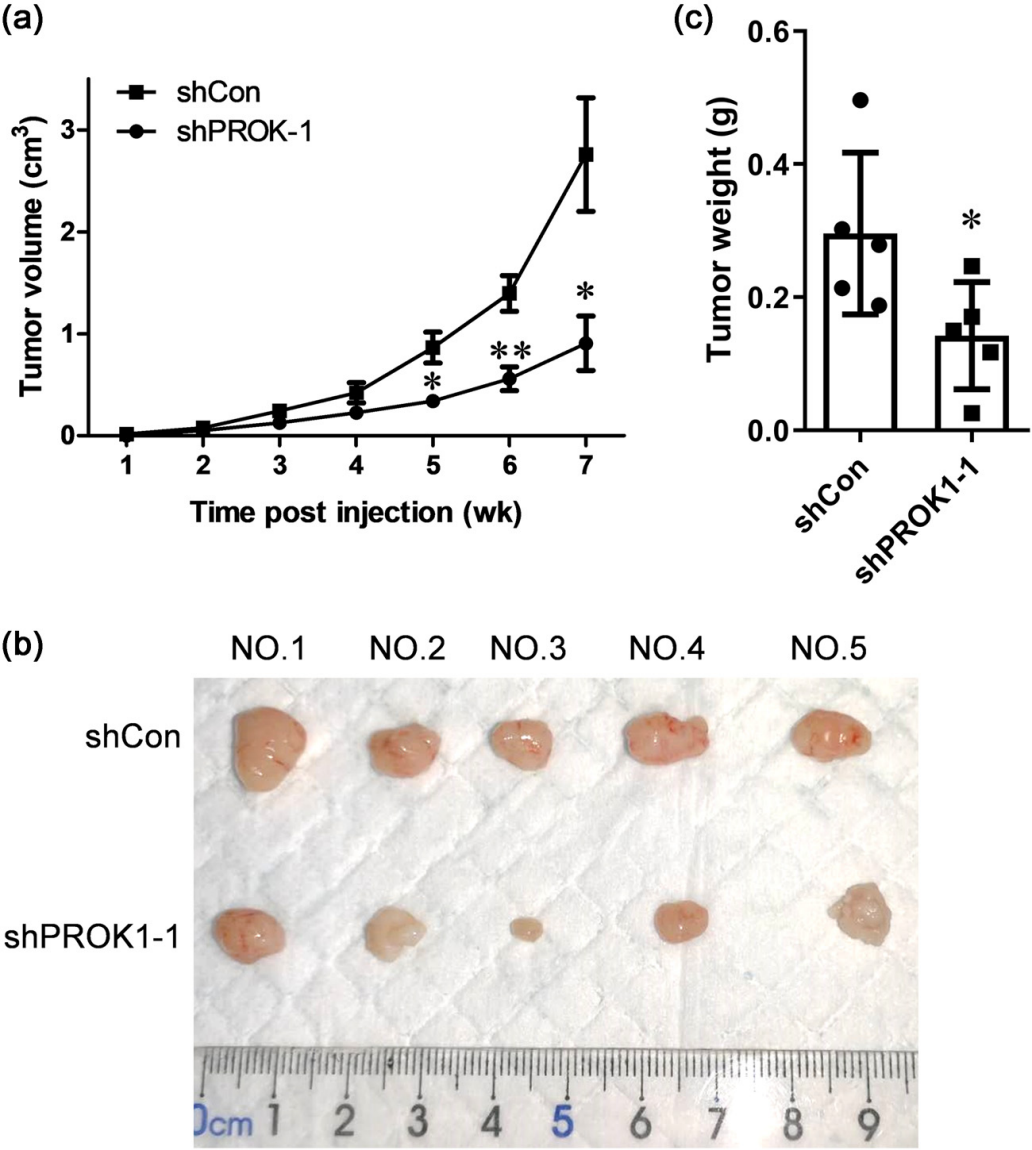
PROK1 knockdown decreased the volume and weight of human PC xenograft in BALB/c nude mice. (a) Tumor volume was measured using calipers every week after injecting cancer cells. Ten mice in the shCon group were injected with 2 × 10^6^ control PANC-1 cells, and the mice in the other group were injected with PANC-1 cells transfected with sh-PROK1–1. (b and c) We measured the size of tumor tissue using calipers and the tumor weight; *P < 0.05 and **P < 0.01, compared to the shCon group.

Tumor volume was measured using calipers every 6 days, after 35 days post-injection. The differences in the tumor volume between the groups increased with time (*P* < 0.01, Figure 1a). The size of the tumor in the sh-PROK1–1 group was smaller than that in the shCon group (Figure 1b). Tumor weight was recorded after harvesting the tissue. The tumor weight of the sh-PROK1–1 group was lesser than that of the shCon group (*P* < 0.01, Figure 1c). These results indicated that PROK1 knockdown significantly inhibited the growth of PC in BALB/c nude mice. Because PROK1 is an angiogenic inducer, vascular characteristic changes could be determined from the results of H&E staining. After histological evaluation of the tissue, we found no other noticeable morphological changes in the blood vessels, but the formation of blood vessels decreased, and more fluid and crowded cells were present in the shPROK1 group than in the shCon group (Figure S1).

### 3.2 PROK1 knockdown inhibited the PI3K/AKT/mTOR pathway and proliferation markers and increased apoptosis

**Figure 2:**
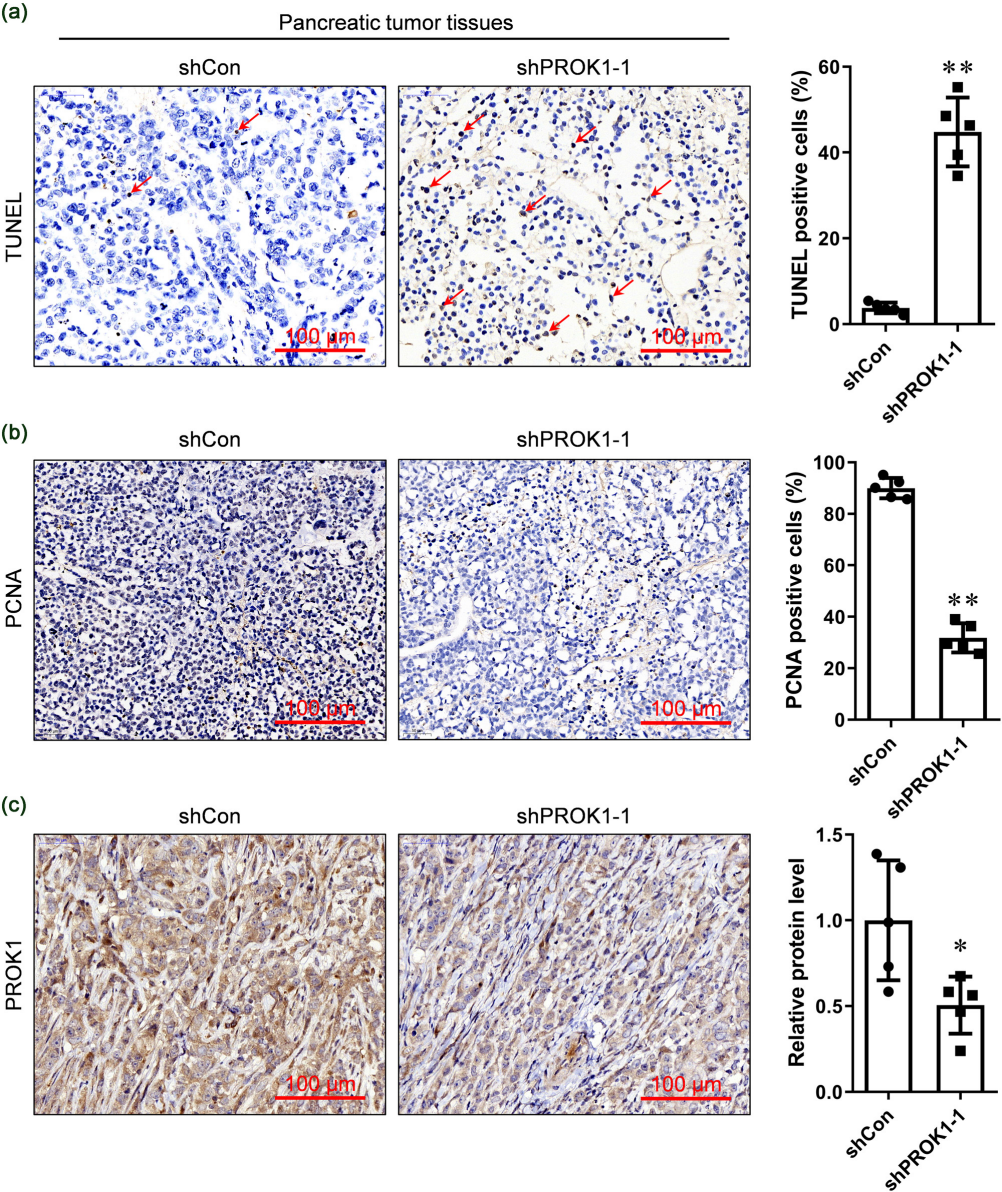
PROK1 silencing increased apoptosis and decreased the expression of PROK1. (a) The TUNEL micrograph of the sh-PROK1–1 group. Multiple brown-stained cancer cells were found in the sh-PROK1–1-treated cells, indicating apoptosis. (b) The cell proliferation marker PCNA was detected by the IHC analysis. (c) The PROK1 protein level in PC tissues was detected by the IHC analysis. Scale bar: 100 µm; *P < 0.05 and **P < 0.01, versus the shCon group.

The TUNEL micrographs of the tumor sliced from the sh-PROK1–1 group showed multiple brown-stained (TUNEL-positive, indicating apoptosis) cancer cells (Figure 2a). After PROK1 knockdown with shRNA, the proliferating marker PCNA-positive stained cells decreased significantly (Figure 2b). The results of the IHC analysis showed that PROK1 was weakly expressed in the shPROK1 group but strongly expressed in the shCon group (Figure 2c). This indicated that treatment with sh-PROK1–1 helped to maintain the reduced expression of PROK1 during tumor growth.

**Figure 3:**
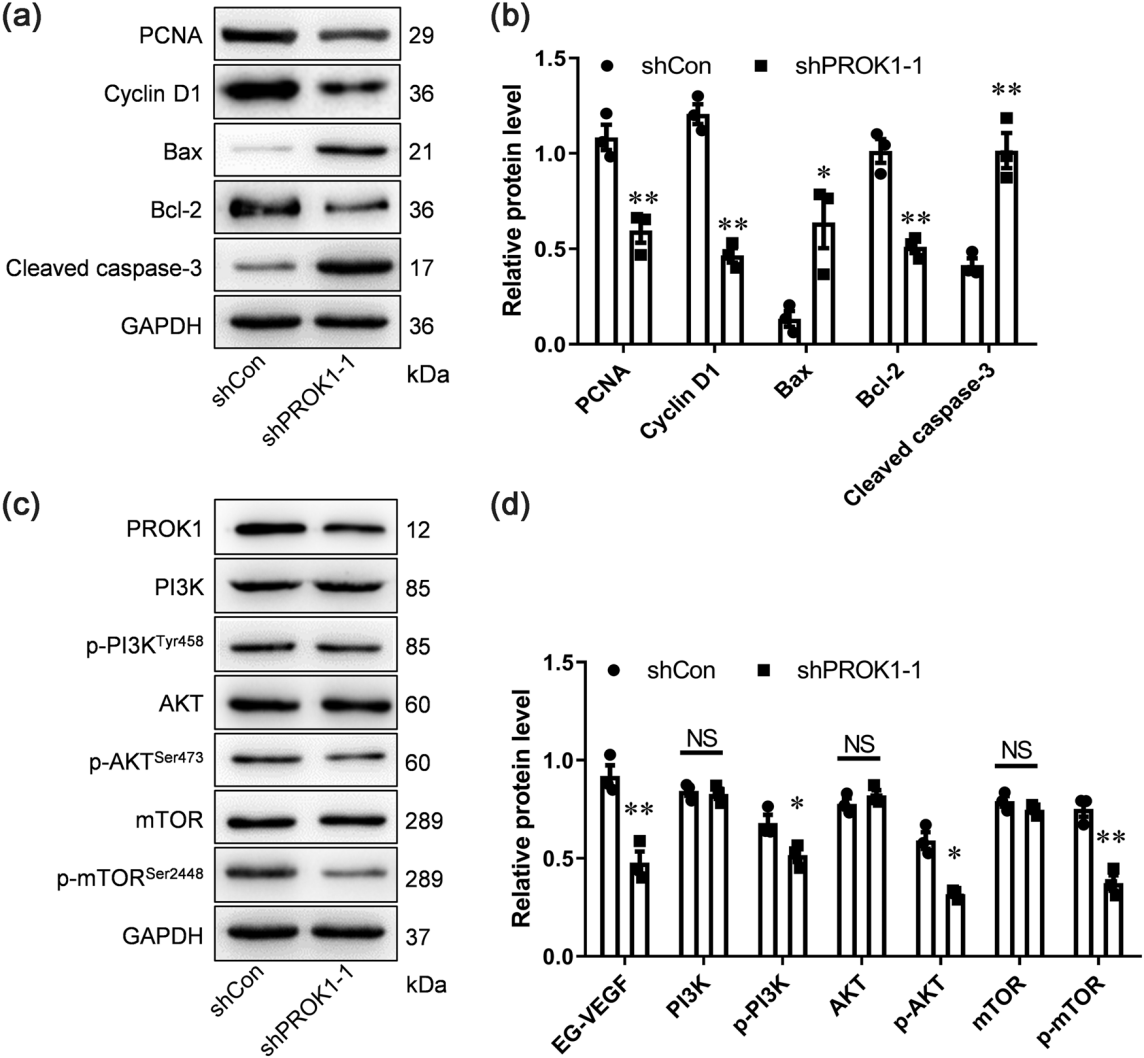
Effect of PROK1 knockdown on proteins involved in the PI3K/AKT/mTOR pathway, proliferation, and apoptosis. (a and b) The protein levels of p-PI3K, PI3K, p-AKT, AKT, mTOR, and p-mTOR in the BALB/c mouse PC xenograft were measured by performing Western blotting, and densitometric analysis was performed using the ImageJ software. (c) Western blotting was performed to measure the relative expression of the PCNA and cyclin D1 proteins (markers expressed only in proliferating cells), Bcl-2 (a marker of anti-apoptosis), and Bax and cleaved caspase-3 (markers of pro-apoptosis). (d) The optical density of each protein was analyzed; *P < 0.05 and **P < 0.01, compared to the shCon group.

The results of the Western blot analysis showed that the protein level of PCNA, cyclin D1, and Bcl-2 was lower in the shPROK1 group than that in the shCon group, while the protein level of Bax and cleaved caspase-3 in the shPROK1 group was higher than that in the other groups (Figure 3a and b). To determine whether the apoptosis of the human PC xenograft cells induced by PROK1 knockdown was related to the inactivation of the PI3K/AKT/mTOR signal, the level of the proteins associated with the pathway was measured. The results showed that the levels of p-PI3K, p-AKT, and p-mTOR were significantly reduced by the knockdown of PROK1 in the BALB/c mouse PC xenograft (*P* < 0.05 and *P* < 0.01 versus the shCon group), suggesting that the inhibition of the PI3K/AKT/mTOR pathway was induced by PROK1 knockdown (Figure 3c and d). To summarize, our results revealed that the inhibition of the phosphorylation of the PI3K/AKT/mTOR mediated by PROK1 knockdown induced cell apoptosis, which might be why PROK1 silencing promoted the human PC xenograft tissues to shrink and the cells in BALB/c nude mice to undergo apoptosis.

### 3.3 The role of PROK1 in PC patient survival

**Figure 4:**
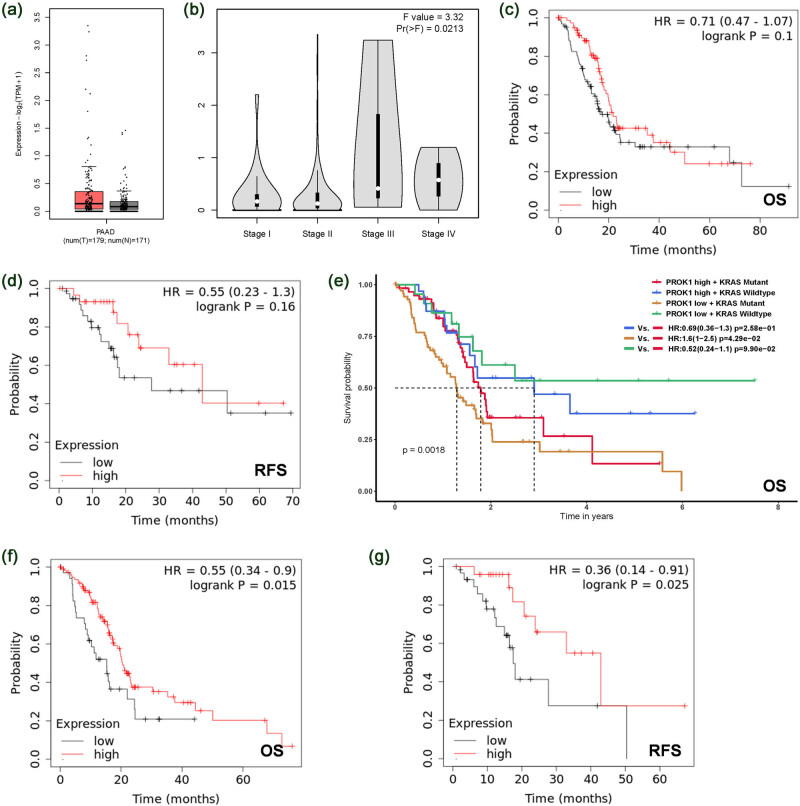
The possible role of PROK1 in the survival of PAAD patients. (a and b) Using public sequencing data in the TCGA and GTEx databases and the online tools GEPIA 2, ToPP, and Kaplan–Meier plotter, the expression level of PROK1 in PAAD was determined. (c–g) The relationship between the expression level of PROK1 and the overall or progression-free survival of the patients was determined.

The difference in the transcription of PROK1 in PAAD tissues and non-PAAD tissues was not significant (Figure 4a and b). Moreover, the expression level significantly increased with the increase in the tumor stage, suggesting a possible role in the progression of PAAD. The level of PROK1 and the overall survival (OS) or relapse-free survival (RFS) of PAAD patients were not associated (Figure 4c and d). Considering that the mutation of some genes like KRAS usually occurs in PAAD, we subgrouped the data with mutated or non-mutated KRAS and reanalyzed the relationship. In patients with mutated KRAS, higher PROK1 had longer RFS (Figure 4e). We also analyzed the relationship in stage 2 patients and found that survival probability (including OS and RFS) increased with the increase in the PROK1 level (Figure 4f and g), which was unexpected.

### 3.4 The possible related molecules of PROK1 and their roles in regulating tumor growth in PC

**Figure 5:**
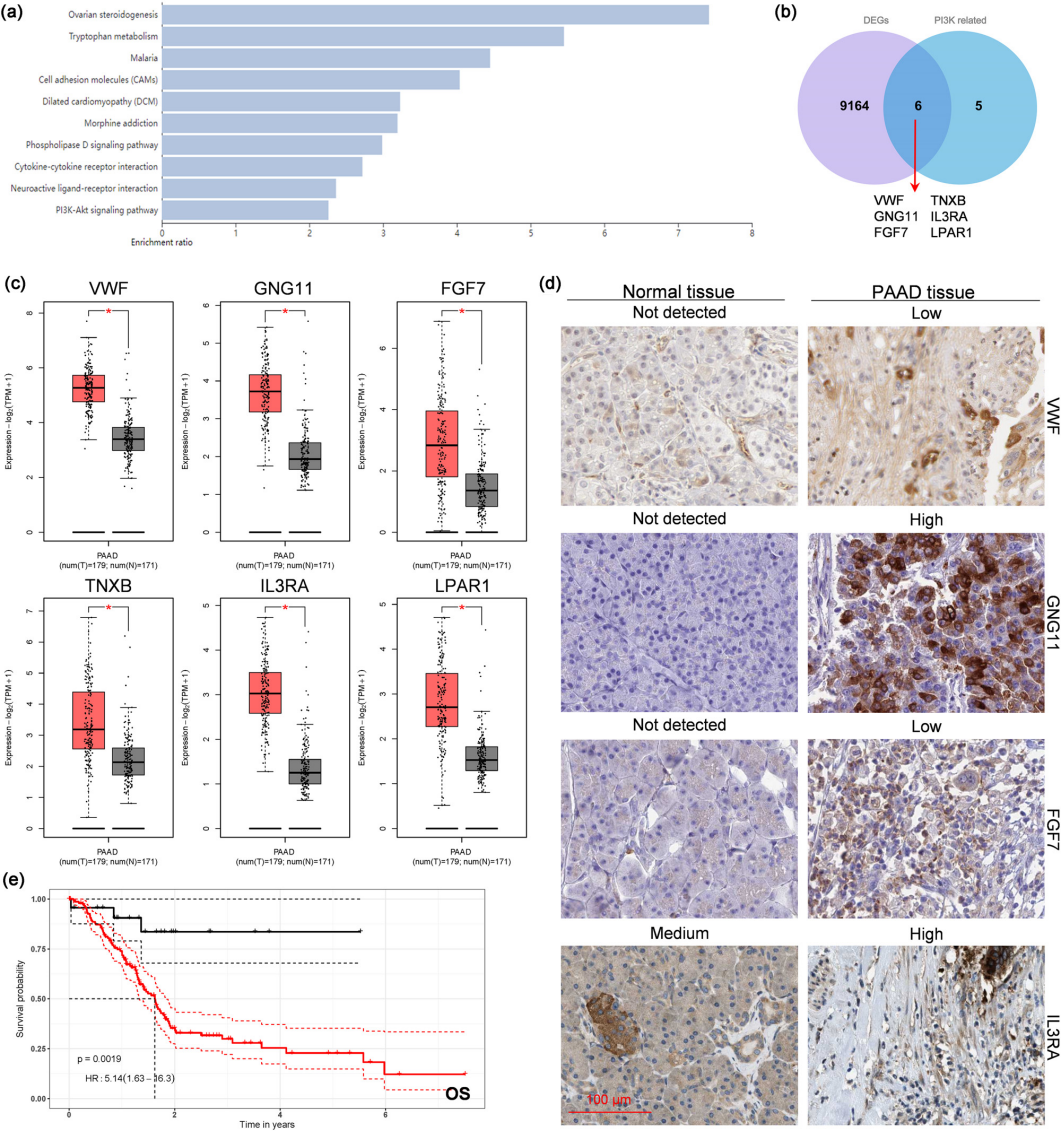
Screening of genes related to PROK1 and their expression and association with survival. (a) The correlated genes from GEPIA 2 were submitted to WebGestalt to perform gene enrichment. (b) The genes involved in PI3K/AKT signaling were overlapped with DEGs. After the intended genes were identified, their mRNA level, protein level, and possible association with survival were analyzed using (c) GEPIA 2, (d) THPA, and (e) ToPP.

The 500 genes correlated with PROK1 in PAAD (Pearson’s correlation, *R* > 0.3) were obtained from the TCGA database using GEPIA 2 and then submitted to WebGestalt for the KEGG pathway enrichment analysis. The results showed that they were mainly enriched in ovarian steroidogenesis, tryptophan metabolism, and malaria. However, several genes (including 11 genes: IGF1, NGFR, VWF, GNG11, NGF, FGF7, TNXB, IL3RA, LPAR1, TCL1B, and GH1) were associated with the activation of PI3K/AKT, which was interesting. These genes were overlapped with 9,170 DEGs in PAAD, which were obtained by comparing the mRNA expression profile between pancreatic cancer in TCGA and the normal pancreas in GTEx; six specific genes were found, including VWF, GNG11, FGF7, TNXB, IL3RA, and LPAR1 (Figure 5a and b). The expression of all six genes was significantly upregulated in PAAD compared to that in non-PAAD (Figure 5c). According to the data on THPA, most of these genes (VWF, GNG11, FGF7, and IL3RA) were also higher in PAAD tissues at the protein level (compared to non-tumor tissues, Figure 5d). The results of the univariate analysis for the association between the interested gene and the survival of the patients showed no notable relationship between them irrespective of whether KRAS was mutated (Figure S2). However, the results of the multivariate analysis showed that the increase in the transcription of these genes was associated with a decrease in the OS of the PAAD patients (Figure 5e).

## 4 Discussion

Our results showed that PROK1 knockdown inhibited pancreatic tumor growth *in vivo* and reduced proliferating markers, such as PCNA and cyclin D1, at the molecular level. It also increased DNA damage and apoptosis markers (cleaved caspase-3 and Bax/Bcl-2 ratio) and inactivated PI3K/Akt/mTOR signaling. PROK1 binds to homologous G protein-coupled receptors, including PROKR1 and PROKR2. The PROK1/PROKR system has potent angiogenic activities in cancer development. In addition to promoting angiogenesis, PROK1 regulates cell migration and proliferation in normal and cancer cells [8,18,19]. PROKR1 expression is positively correlated with its ligand in trophoblasts that exhibit a “pseudo-tumorigenesis” feature [20,21], and PROKR1 mediates the proliferation of neuroblastoma cells [22]. Therefore, PROK1–PROKR1 interaction might be the key process by which PROK1 induces proliferation. In our study, the expression of PCNA and cyclin D1 proteins was considerably lower in the treated group than in the shCon group, suggesting that the downregulation of PROK1 inhibited the proliferation of human PC xenografts in nude mice. This provided additional evidence for the proliferative effects of PROK1 in cancer patients. The angiogenic and proliferative effects of PROK1 might contribute to poorer cancer-related survival in patients with pancreatic cancer and colorectal cancer [23].

Apoptosis, a type of classical programmed cell death, is an active, procedural, and intrinsic biological process characterized by a genetically controlled cell-autonomous death pattern. The unrestricted growth of tumors occurs due to the inhibition of tumor cell apoptosis. Thus, the apoptotic disorder is closely related to the occurrence, development, and malignant transformation of tumors [24]. Bcl-2 and Bax are closely related to apoptosis and belong to the Bcl-2 superfamily. These proteins have anti-apoptotic and pro-apoptotic effects, respectively [25]. When the Bax/Bcl-2 ratio increases, excess Bax proteins form homodimers and activate apoptosis, but when the ratio decreases, Bax-Bcl-2 heterodimer formation elicits a survival signal for both normal and tumor cells [26]. Caspase-3 belongs to the caspase family and is a very important protease that performs apoptosis, and it is activated at the end of the caspase cascade [27] through the successive cleavage of the interdomain linker and N-terminal prodomain. Its cleaved form is necessary for apoptotic chromatin condensation and DNA fragmentation [28,29]. To some extent, the level of expression of the caspase-3 gene determines how much caspase-3 can function [30]. We found in this study that the protein level of Bcl-2 decreased and that of Bax and cleaved caspase-3 increased, indicating that PROK1 silencing promoted the apoptotic death of tumor cells transplanted in nude mice. Our findings were similar to those of previous studies that showed PROK-1 induced Akt phosphorylation, upregulated the Bcl-2 family member Mcl-1, and acted as an anti-apoptotic effector in cancer cells [31,32].

Apoptosis is usually induced by intracellular pathways and/or signals outside cells [33]. The PI3K/AKT/mTOR pathway is important in cells and regulates various cellular activities, including proliferation and apoptosis [34]. However, its main function is to promote cell proliferation, survival, and the cell cycle and also participate in angiogenesis [35]. The PI3K/AKT/mTOR pathway can be activated by PROK1, which is associated with almost all human cancers [22,36]. Our experimental results and data mining results showed that PROK1 could also regulate the PI3K/AKT pathway, suggesting that the downregulation of PROK1 can inhibit PC cells, in which the PI3K/AKT/mTOR pathway probably participates.

Through data mining in the public databases, we found six genes, including VWF, GNG11, FGF7, TNXB, IL3RA, and LPAR1, which might be associated with the PI3K/AKT signaling pathway. Although the individual expression of these genes was not associated with patient survival, the combined tumor-promoting effect of these genes on PAAD needs further investigation as it might reveal their mechanism of action. Additionally, these genes were shown by other researchers to play important roles in cancer. For example, VWF inhibits bleeding, and its level is elevated in PC, which might be the reason for the development of thrombosis in these patients [37]. An increase in VWF might enhance the metastatic activity of pancreatic cancer and result in a poorer prognosis [38]. GNG11 participates in EMT and strongly regulates cellular senescence. The amplification of its gene leads to the activation of the Ras/Raf/MEK pathway in this tumor with wild-type RAS and RAF genes [39,40,41]. FGF7 and TNXB (tenascin X plays a role in organizing and maintaining the structure of muscle tissues, connective tissues, etc., by producing and assembling certain types of collagen) can increase pancreatic cancer cell migration and invasion [42,43,44]. The expression of TNXB is downregulated in some pancreatic cell lines [45], and it is correlated with a good survival prognosis in pan-cancer [46]. IL3RA (CD123) encodes a subunit of a receptor for IL-3. It plays a dual role in the immune system and promotes vessel formation [47]. High levels of IL3RA were found in pancreatic ductal adenocarcinoma patients with improved survival [48]. Lysophosphatidic acid receptor 1 (LPAR1) not only regulates the development of intratumoral heterogeneity [49] but also is crucial for chemotaxis and dissemination of pancreatic cancer cells [50]. TNXB and LPAR1 are also associated with activated PI3K/AKT [43,49]. The above-mentioned findings suggest that the final survival results are influenced by many factors, such as motility, heterogeneity, and the surrounding environment (including immune cells and blood vessels) of cancer cells rather than the expression of DEGs in cancer cells. Along with the results of these above-mentioned studies, our findings might provide new insights into the pathogenesis of pancreatic cancer and its treatment.

Our study had some limitations. First, non-homogeneous distribution of blood vessels occurs widely in tumors, and stereoscopy analysis based on serial sections is necessary to evaluate angiogenesis. However, we did not have the sample tissues sectioned serially, and thus, we could only make descriptive and qualitative statements about the representative H&E stained images. Also, the six related genes, such as VMW, might be important for evaluating the effects of PROK-1 knockdown in pancreatic cancer cells, and further experiments were required to elucidate their role. However, due to limited time and budget, they were neglected. We aim to investigate these molecules in follow-up studies.

## 5 Conclusion

To summarize, we found that PROK1 knockdown promoted cell apoptosis, which was characterized by an increase in TUNEL-positive staining, Bax protein levels, and caspase-3 cleavage. PROK1 knockdown also inhibited the proliferation of pancreatic cancer cells, characterized by a decrease in PCNA and cyclin D1 levels, leading to slow growth of PC *in vivo*. Furthermore, a decrease in the PROK1 levels inactivated the PI3K/AKT/mTOR pathway along with the associated molecules, which probably involves VWF, GNG11, FGF7, TNXB, IL3RA, and LPAR1. Therefore, PROK1 might be a potential molecular target for pancreatic cancer therapy, and further studies on it, along with its related genes, might be valuable for treating pancreatic cancer.

## Supplementary Material

Supplementary Figure

## References

[1] Sung H, Ferlay J, Siegel RL, Laversanne M, Soerjomataram I, Jemal A, et al. Global cancer statistics 2020: GLOBOCAN estimates of incidence and mortality worldwide for 36 cancers in 185 countries. CA Cancer J Clin. 2021;71(3):209–49. 10.3322/caac.21660 33538338

[2] Wu LM, Zhang LL, Chen XH, Zheng SS. Is irreversible electroporation safe and effective in the treatment of hepatobiliary and pancreatic cancers? Hepatobiliary Pancreat Dis Int. 2019;18(2):117–24. 10.1016/j.hbpd.2019.01.001 30655073

[3] Rhee H, Park MS. The role of imaging in current treatment strategies for pancreatic adenocarcinoma. Korean J Radiol. 2021;22(1):23–40. 10.3348/kjr.2019.0862 PMC7772381 32901458

[4] Wang D, Rodriguez EA, Barkin JS, Donath EM, Pakravan AS. Statin use shows increased overall survival in patients diagnosed with pancreatic cancer: A meta-analysis. Pancreas. 2019;48(4):e22–3. 10.1097/MPA.0000000000001276 30973465

[5] Melincovici CS, Bosca AB, Susman S, Marginean M, Mihu C, Istrate M, et al. Vascular endothelial growth factor (VEGF)-key factor in normal and pathological angiogenesis. Rom J Morphol Embryol. 2018;59(2):455–67. 30173249

[6] Corlan AS, Cîmpean AM, Jitariu AA, Melnic E, Raica M. Endocrine gland-derived vascular endothelial growth Factor/Prokineticin-1 in cancer development and tumor angiogenesis. Int J Endocrinol. 2017;2017:3232905. 10.1155/2017/3232905 PMC5366234 28386275

[7] Jiang X, Abiatari I, Kong B, Erkan M, De Oliveira T, Giese NA, et al. Pancreatic islet and stellate cells are the main sources of endocrine gland-derived vascular endothelial growth factor/prokineticin-1 in pancreatic cancer. Pancreatology. 2009;9(1):165–72. 10.1159/000178888 19077468

[8] Yan X, Hui Y, Hua Y, Huang L, Wang L, Peng F, et al. EG-VEGF silencing inhibits cell proliferation and promotes cell apoptosis in pancreatic carcinoma via PI3K/AKT/mTOR signaling pathway. Biomed Pharmacother. 2019;109:762–9. 10.1016/j.biopha.2018.10.125 30551529

[9] Yu L, Wei J, Liu P. Attacking the PI3K/Akt/mTOR signaling pathway for targeted therapeutic treatment in human cancer. Semin Cancer Biol. 2022;85:69–94. 10.1016/j.semcancer.2021.06.019 34175443

[10] Lien EC, Dibble CC, Toker A. PI3K signaling in cancer: Beyond AKT. Curr Opin Cell Biol. 2017;45:62–71. 10.1016/j.ceb.2017.02.007 PMC5482768 28343126

[11] Zhao L, Vogt PK. Class I PI3K in oncogenic cellular transformation. Oncogene. 2008;27(41):5486–96. 10.1038/onc.2008.244 PMC2757120 18794883

[12] Zhu YP, Brown JR, Sag D, Zhang L, Suttles J. Adenosine 5′-monophosphate-activated protein kinase regulates IL-10-mediated anti-inflammatory signaling pathways in macrophages. J Immunol. 2015;194(2):584–94. 10.4049/jimmunol.1401024 PMC4343033 25512602

[13] Jin S, Borkhuu O, Bao W, Yang YT. Signaling pathways in thyroid cancer and their therapeutic implications. J Clin Med Res. 2016;8(4):284–96. 10.14740/jocmr2480w PMC4780491 26985248

[14] Zou Z, Tao T, Li H, Zhu X. mTOR signaling pathway and mTOR inhibitors in cancer: progress and challenges. Cell Biosci. 2020;10:31. 10.1186/s13578-020-00396-1 PMC7063815 32175074

[15] Zhang Y, Cheng H, Li W, Wu H, Yang Y. Highly-expressed P2X7 receptor promotes growth and metastasis of human HOS/MNNG osteosarcoma cells via PI3K/Akt/GSK3β/β-catenin and mTOR/HIF1α/VEGF signaling. Int J Cancer. 2019;145(4):1068–82. 10.1002/ijc.32207 PMC6618011 30761524

[16] LoPiccolo J, Blumenthal GM, Bernstein WB, Dennis PA. Targeting the PI3K/Akt/mTOR pathway: Effective combinations and clinical considerations. Drug Resist Updat. 2008;11(1–2):32–50. 10.1016/j.drup.2007.11.003 PMC2442829 18166498

[17] Wolpin BM, Hezel AF, Abrams T, Blaszkowsky LS, Meyerhardt JA, Chan JA, et al. Oral mTOR inhibitor everolimus in patients with gemcitabine-refractory metastatic pancreatic cancer. J Clin Oncol. 2009;27(2):193–8. 10.1200/JCO.2008.18.9514 PMC2645085 19047305

[18] Goryszewska-Szczurek E, Baryla M, Kaczynski P, Waclawik A. Prokineticin 1-prokineticin receptor 1 signaling in trophoblast promotes embryo implantation and placenta development. Sci Rep. 2021;11(1):13715. 10.1038/s41598-021-93102-1 PMC8253840 34215801

[19] Goi T, Nakazawa T, Hirono Y, Yamaguchi A. The anti-tumor effect is enhanced by simultaneously targeting VEGF and PROK1 in colorectal cancer. Oncotarget. 2015;6(8):6053–61. 10.18632/oncotarget.3474 PMC4467421 25788276

[20] Soundararajan R, Rao AJ. Trophoblast ‘pseudo-tumorigenesis’: significance and contributory factors. Reprod Biol Endocrinol. 2004;2:15. 10.1186/1477-7827-2-15 PMC407853 15043753

[21] Kisliouk T, Friedman A, Klipper E, Zhou QY, Schams D, Alfaidy N, et al. Expression pattern of prokineticin 1 and its receptors in bovine ovaries during the estrous cycle: involvement in corpus luteum regression and follicular atresia. Biol Reprod. 2007;76(5):749–58. 10.1095/biolreprod.106.054734 17229935

[22] Ngan ES, Sit FY, Lee K, Miao X, Yuan Z, Wang W, et al. Implications of endocrine gland-derived vascular endothelial growth factor/prokineticin-1 signaling in human neuroblastoma progression. Clin Cancer Res. 2007;13(3):868–75. 10.1158/1078-0432.CCR-06-2176 17289879

[23] Tagai N, Goi T, Shimada M, Kurebayashi H. Plasma Prokineticin 1, a prognostic biomarker in colorectal cancer patients with curative resection: a retrospective cohort study. World J Surg Oncol. 2021;19(1):302. 10.1186/s12957-021-02421-0 PMC8522247 34657605

[24] Arora J, Sauer SJ, Tarpley M, Vermeulen P, Rypens C, Van Laere S, et al. Inflammatory breast cancer tumor emboli express high levels of anti-apoptotic proteins: Use of a quantitative high content and high-throughput 3D IBC spheroid assay to identify targeting strategies. Oncotarget. 2017;8(16):25848. 10.18632/oncotarget.15667 PMC5432221 28460441

[25] Xu B, Lian S, Guo JR, Wang JF, Zhang LP, Li SZ, et al. Activation of the MAPK signaling pathway induces upregulation of pro-apoptotic proteins in the hippocampi of cold stressed adolescent mice. Neurosci Lett. 2019;699:97–102. 10.1016/j.neulet.2018.12.028 30711527

[26] Basu A, Haldar S. The relationship between BcI2, Bax and p53: consequences for cell cycle progression and cell death. Mol Hum Reprod. 1998;4(12):1099–109. 10.1093/molehr/4.12.1099 9872359

[27] Wang KS, Chan CK, Hidayat AFA, Wong YH, Kadir HA. Clinacanthus nutans induced reactive oxygen species-dependent apoptosis and autophagy in HCT116 human colorectal cancer cells. Pharmacogn Mag. 2019;15(60):87.

[28] Porter AG, Jänicke RU. Emerging roles of caspase-3 in apoptosis. Cell Death Differ. 1999;6(2):99–104. 10.1038/sj.cdd.4400476 10200555

[29] Ponder KG, Boise LH. The prodomain of caspase-3 regulates its own removal and caspase activation. Cell Death Discov. 2019;5:56. 10.1038/s41420-019-0142-1 PMC6349851 30701088

[30] Huang TC, Chiu PR, Chang WT, Hsieh BS, Huang YC, Cheng HL, et al. Epirubicin induces apoptosis in osteoblasts through death-receptor and mitochondrial pathways. Apoptosis. 2018;23(3–4):226–36. 10.1007/s10495-018-1450-2 29468482

[31] Shi JF, Lu BL, Huang B, Mao R, Tang JY, Zhu XY. Research progress of an novel vascular endothelial growth factor EG-VEGF/PROK1 in tumors. J Mod Oncol. 2018;22:3682–6.

[32] Ren LN, Li QF, Xiao FJ, Yan J, Yang YF, Wang LS, et al. Endocrine glands-derived vascular endothelial growth factor protects pancreatic cancer cells from apoptosis via upregulation of the myeloid cell leukemia-1 protein. Biochem Biophys Res Commun. 2009;386(1):35–9. 10.1016/j.bbrc.2009.05.149 19523441

[33] Mi Y, Xiao C, Du Q, Wu W, Qi G, Liu X. Momordin Ic couples apoptosis with autophagy in human hepatoblastoma cancer cells by reactive oxygen species (ROS)-mediated PI3K/Akt and MAPK signaling pathways. Free Radic Biol Med. 2016;90:230–42. 10.1016/j.freeradbiomed.2015.11.022 26593748

[34] Granato M, Rizzello C, Gilardini Montani MS, Cuomo L, Vitillo M, Santarelli R, et al. Quercetin induces apoptosis and autophagy in primary effusion lymphoma cells by inhibiting PI3K/AKT/mTOR and STAT3 signaling pathways. J Nutr Biochem. 2017;41:124–36. 10.1016/j.jnutbio.2016.12.011 28092744

[35] Wang SS, Chen YH, Chen N, Wang LJ, Chen DX, Weng HL, et al. Hydrogen sulfide promotes autophagy of hepatocellular carcinoma cells through the PI3K/Akt/mTOR signaling pathway. Cell Death Dis. 2017;8(3):e2688. 10.1038/cddis.2017.18 PMC5386547 28333142

[36] Saini S, Maker AV, Burman KD, Prabhakar BS. Molecular aberrations and signaling cascades implicated in the pathogenesis of anaplastic thyroid cancer. Biochim Biophys Acta Rev Cancer. 2019;1872(2):188262. 10.1016/j.bbcan.2018.12.003 30605717

[37] Markocka-Mączka K. Von Willebrand factor (vWF) in plasma of patients with pancreatic carcinoma. Contemp Oncol. 2002;6(5):322–6.

[38] Patmore S, Dhami SPS, O’Sullivan JM. Von Willebrand factor and cancer; metastasis and coagulopathies. J Thromb Haemost. 2020;18(10):2444–56. 10.1111/jth.14976 32573945

[39] Kosr MA, Ju D. The CXCL7/CXCR2 axis and the migration of breast cells toward the malignant phenotype. J Clin Oncol. 2012;30(27_suppl):181.

[40] Miwa T, Kanda M, Tanaka H, Tanaka C, Kobayashi D, Umeda S, et al. FBXO50 enhances the malignant behavior of gastric cancer cells. Ann Surg Oncol. 2017;24(12):3771–9. 10.1245/s10434-017-5882-7 28560594

[41] Hossain MN, Sakemura R, Fujii M, Ayusawa D. G-protein gamma subunit GNG11 strongly regulates cellular senescence. Biochem Biophys Res Commun. 2006;351(3):645–50. 10.1016/j.bbrc.2006.10.112 17092487

[42] Huang T, Wang L, Liu D, Li P, Xiong H, Zhuang L, et al. FGF7/FGFR2 signal promotes invasion and migration in human gastric cancer through upregulation of thrombospondin-1. Int J Oncol. 2017;50(5):1501–12. 10.3892/ijo.2017.3927 PMC5403236 28339036

[43] Yan SP, Chu DX, Qiu HF, Xie Y, Wang CF, Zhang JY, et al. LncRNA LINC01305 silencing inhibits cell epithelial-mesenchymal transition in cervical cancer by inhibiting TNXB-mediated PI3K/Akt signalling pathway. J Cell Mol Med. 2019;23(4):2656–66. 10.1111/jcmm.14161 PMC6433725 30697971

[44] Carter EP, Coetzee AS, Tomas Bort E, Wang Q, Kocher HM, Grose RP. Dissecting FGF signalling to target cellular crosstalk in pancreatic cancer. Cells. 2021;10(4):847. 10.3390/cells10040847 PMC8068358 33918004

[45] Shimizu H, Horii A, Sunamura M, Motoi F, Egawa S, Unno M, et al. Identification of epigenetically silenced genes in human pancreatic cancer by a novel method “microarray coupled with methyl-CpG targeted transcriptional activation” (MeTA-array). Biochem Biophys Res Commun. 2011;411(1):162–7. 10.1016/j.bbrc.2011.06.121 21723258

[46] Liot S, Aubert A, Hervieu V, Kholti NE, Schalkwijk J, Verrier B, et al. Loss of Tenascin-X expression during tumor progression: A new pan-cancer marker. Matrix Biol Plus. 2020;6–7:100021. 10.1016/j.mbplus.2020.100021 PMC7852205 33543019

[47] Lombardo G, Gili M, Grange C, Cavallari C, Dentelli P, Togliatto G, et al. IL-3R-alpha blockade inhibits tumor endothelial cell-derived extracellular vesicle (EV)-mediated vessel formation by targeting the β-catenin pathway. Oncogene. 2018;37(9):1175–91. 10.1038/s41388-017-0034-x PMC5861089 29238040

[48] Boucher Y, Posada JM, Subudhi S, Rosario SR, Gu L, Kumar AS, et al. Addition of losartan to FOLFORINOX and chemoradiation downregulates pro-invasion and immunosuppression-associated genes in locally advanced pancreatic cancer. medRxiv. 2022.06.09.22275912. 2022. 10.1101/2022.06.09.22275912. PMC10106451 36749873

[49] Cui R, Cao G, Bai H, Zhang Z. LPAR1 regulates the development of intratumoral heterogeneity in ovarian serous cystadenocarcinoma by activating the PI3K/AKT signaling pathway. Cancer Cell Int. 2019;19(1):201. 10.1186/s12935-019-0920-0 PMC6664705 31384176

[50] Juin A, Spence HJ, Martin KJ, McGhee E, Neilson M, Cutiongco MFA, et al. N-WASP control of LPAR1 trafficking establishes response to self-generated LPA gradients to promote pancreatic cancer cell metastasis. Dev Cell. 2019;51(4):431–45.e7. 10.1016/j.devcel.2019.09.018 PMC6863394 31668663

