# Supplementary material for: PI3K/AKT/mTOR pathway and its related molecules participate in PROK1 silence-induced anti-tumor effects on pancreatic cancer
Source: Open Life Sci. 2023 Apr 10;18(1):20220538. doi: 10.1515/biol-2022-0538 (PMC10105552; doi:10.1515/biol-2022-0538)
Supplement: Supplementary Figure [file biol-2022-0538-sm.pdf]

Supplementary material

Figures S1 and S2

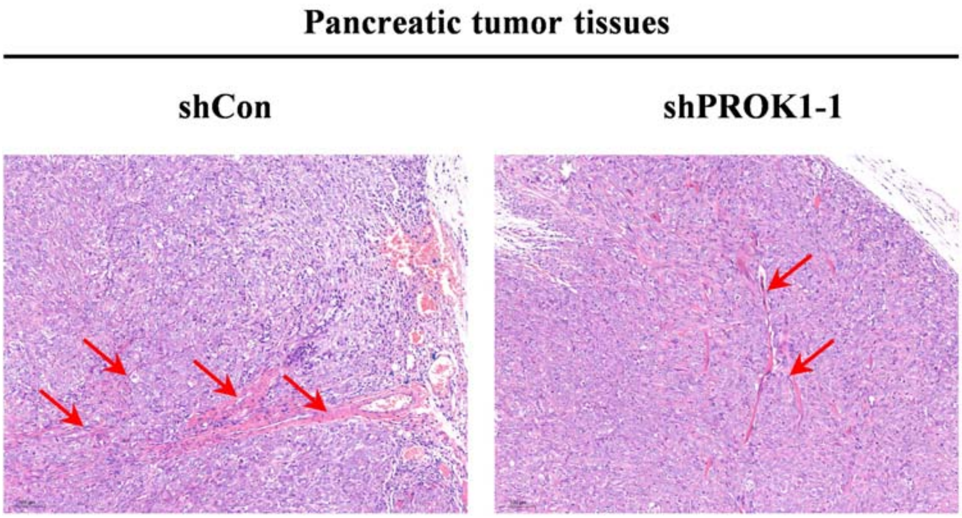

Figure S1: Effects of PROKl knockdown on vessel features of pancreatic tumor. Vascular characteristics were observed through H&E staining. The histological evaluation of tissue showed the formation of blood vessels was inhibited in shPROKl group (versus shCon group).

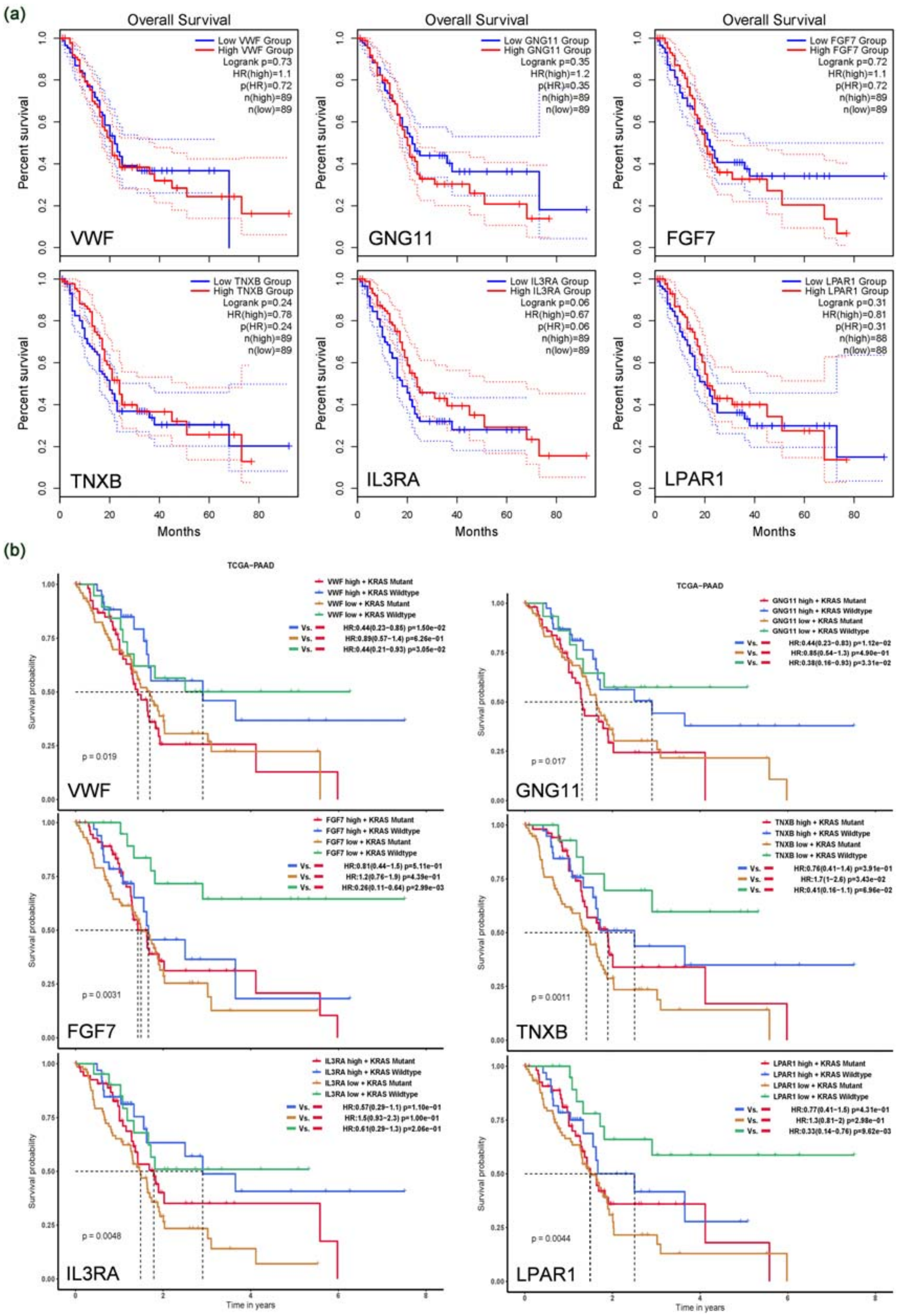

Figure S2: Possible related genes of PROKl were not in association with patient survival. The association between VWF, GNG11, FGF7, TNXB, IL3RA, or LPARl and OS in all PAAD patients was analyzed with Kaplan Meier plotter (a), and the association in PADD patients with KRAS mutation was analyzed using ToPP (b).
